# A systematic review of the impact of parental socio-economic status and home environment characteristics on children’s oral health related quality of life

**DOI:** 10.1186/1477-7525-12-41

**Published:** 2014-03-21

**Authors:** Santhosh Kumar, Jeroen Kroon, Ratilal Lalloo

**Affiliations:** 1 Population & Social Health Research Program, Griffith Health Institute, School of Dentistry and Oral Health, Gold Coast, Australia

**Keywords:** Oral health related quality of life, Children, Socio-economic status, Home environment

## Abstract

Childhood circumstances such as socio-economic status and family structure have been found to influence psychological, psychosocial attributes and Oral Health Related Quality of Life (OHRQoL) in children. Therefore, the aim of this study was to conduct a systematic review of the published literature to assess the influence of parental Socio-Economic Status (SES) and home environment on children’s OHRQoL. A systematic search was conducted in August 2013 using PubMed, Medline via OVID, CINAHL Plus via EBSCO, and Cochrane databases. Studies that have analysed the effect of parental characteristics (SES, family environment, family structure, number of siblings, household crowding, parents’ age, and parents’ oral health literacy) on children’s OHRQoL were included. Quality assessment of the articles was done by the Effective Public Health Practice Project’s Quality Assessment Tool for Quantitative studies. Database search retrieved a total of 2,849 titles after removing the duplicates, 36 articles were found to be relevant. Most of the studies were conducted on Brazilian children and were published in recent two years. Early Childhood Oral Health Impact Scale and Children’s Perception Questionnaire_11-14_ were the instruments of choice in preschool and school aged children respectively. Findings from majority of the studies suggest that the children from families with high income, parental education and family economy had better OHRQoL. Mothers’ age, family structure, household crowding and presence of siblings were significant predictors of children’s OHRQoL. However, definitive conclusions from the studies reviewed are not possible due to the differences in the study population, parental characteristics considered, methods used and statistical tests performed.

## Introduction

The World Health Organisation (WHO) defines Quality of Life (QoL) as “an individual’s perception of their position in life in the context of the cultural and value systems in which they live and in relation to their goals, expectations, standards and concerns” [1]. Currently, there is a growing interest and move towards the use of patient-focussed assessments to gain more meaningful information, although subjective, on the impact of oral disease on an individual [2]. This is because clinical indicators alone do not reveal the full impact of oral conditions on the psychosocial wellbeing of a patient [3]. Thus, it has been proposed that an evaluation of physical functioning and psychological wellbeing should be complemented with a normative oral-health assessment [4].

Previously concerns were raised that children’s reports of their health and QoL would not meet accepted psychometric standards of validity and reliability, because of limitations in their cognitive capacities and communication skills, [5-7] but currently several validated Oral Health Related Quality of Life (OHRQoL) instruments are aimed at school-aged children [6-9] and preschool children [10,11].

Studies show that children’s oral-health status is often related to social dimensions, such as parental income and education [12]. Furthermore, childhood circumstances, as indicated by socio-economic status (SES), family structure and parenting quality, have been found to influence psychological and psychosocial attributes in children [13]. This is strengthened by findings from recent studies where parental socio-economic factors as well as home environment have been found to impact negatively on children’s OHRQoL [14], with children residing in orphanages presenting with poorer OHRQoL than those living with their parents [15]. However, this is not always the case, with conflicting findings from a few studies where parental SES and home environment characteristics were found to be insignificant in predicting children’s OHRQoL.

Determining the intervening variables that mediate the relationships between clinical variables and OHRQoL will facilitate the design of optimally effective clinical interventions [16]. While a systematic review has been conducted on the association of children’s oral health status with their OHRQoL [17], there is currently no published evidence available on the influence of parental attributes on children’s OHRQoL. Therefore, the aim of this study was to conduct a systematic review of the published literature to assess the influence of parental SES and home environment on children’s OHRQoL.

## Methods

### Search criteria

The protocol for this systematic review was registered with the International Prospective Register of Systematic Reviews, and allocated with the registration number CRD42013005433. The Preferred Reporting Items for Systematic Reviews and Meta-Analyses (PRISMA) guidelines for conducting a systematic review were used [18]. A search for eligible journal articles was undertaken in August 2013, using PubMed, Medline via OVID, CINAHL Plus via EBSCO, and Cochrane databases to answer if parental characteristics (SES, family environment, family structure, number of siblings, household crowding, parents’ age, and parents’ oral health literacy) influence children’s OHRQoL. The search strategy that was used in PubMed is presented in Table 1. In order to prevent the loss of potential articles, a broad range of Medical Subject Heading (MeSH) terms and combination of search strategies were used. As “Oral Health Related Quality of Life” is not a MeSH term, it was used as a keyword to search in all the fields. A truncation for the MesH term “child” was used, as the search term “child” could have many variants. For parental characteristics, a wide-ranging list of subject headings and subheadings were used that were related to “socio-economic status” and “home environment”. In PUBMED, there was no time limit set in the search criteria, while the lower limit for entry date in Medline via OVID and CINAHL Plus via EBSCO was set to 1946 and 1997 respectively. Titles from all languages were considered, since a few journals publish articles both in English and foreign languages.

**Table 1:** Search strategy used in PubMed


#1	Oral health related quality of life
#2	(“Child*”[MeSH] OR “Adolescent”[MeSH])
#3	“Oral health”[MeSH]
#4	“Quality of life”[MeSH]
#5	(“Socioeconomic Factors”[MeSH] OR “Social Class”[MeSH] OR “Social Environment”[MeSH] OR “Poverty”[MeSH] OR “Illiteracy”[MeSH] OR “Literacy”[MeSH] OR “Educational Status”[MeSH] OR “Employment”[MeSH] OR “Family Characteristics”[MeSH] OR “Income”[MeSH] OR “Occupations and Professions”[MeSH] OR “Unemployment”[MeSH] OR “Social Change”[MeSH] OR “Family Characteristics”[MeSH] OR “Marital Status”[MeSH] OR “Parenthood”[MeSH] OR “Family Relations”[MeSH] OR “Nuclear Family”[MeSH] OR “Family Functioning”[MeSH] OR “Age Factors”[MeSH] OR “Birth Place”[MeSH] OR “Birth Intervals”[MeSH] OR “Birth Order”[MeSH] OR “Race Factors”[MeSH] OR “Special Populations”[MeSH])
#6	(#1 AND #2)
#7	(#1 AND #2 AND #5)
#8	(#2 AND #3 AND #4)
#9	(#2 AND #3 AND #4 AND #5)

### Study selection and data extraction

All titles retrieved were exported to EndNote (version X6) software, and one of the authors (SK) selected titles that were relevant to OHRQoL in children. The selection criteria for inclusion after reviewing the full text of the articles were as follows:

•the article used validated OHRQoL instruments to assess OHRQoL in children; and

•the study evaluated the influence of SES, family income, family economy, parental occupation, parent’s education level, parent’s demographics, dental health literacy of the caregivers, household crowding, number of siblings, family structure and any other parent-related characteristics on children’s OHRQoL.

Studies were excluded when individuals studied were older than 18 years of age, where full text was not available in English, and if the study did not consider the effect of relevant parental characteristics on OHRQoL of children. Articles that assessed the association of children’s OHRQoL with other variables that are not parent-related, such as ethnicity, geographic location of residence, urbanisation and dental care experienced, were also excluded, as were studies with subjects ranging from children to adults that have studied the association of subject’s SES with OHRQoL but not of the parents. Piloted forms were used by one of the authors (SK) to extract information from each full-text article, which were then screened independently by the other two senior authors for accuracy. Consensus was reached through discussion between the authors where discrepancies occurred.

### Quality assessment of selected articles

The Effective Public Health Practice Project’s (EPHPP) Quality Assessment Tool for Quantitative Studies was used to evaluate the quality of included articles [19]. The EPHPP tool was created primarily for quality assessment of observational and clinical studies based on populations. EPHPP quality assessment involves rating each article on a three-point scale (strong, moderate and weak) in six components: selection bias, study design, confounders, blinding, data-collection methods, and withdrawals and drop-outs. Based on the rating of each methodological component, a global rating of strong, moderate or weak was allocated to each article [20].

## Results

**Figure 1:**
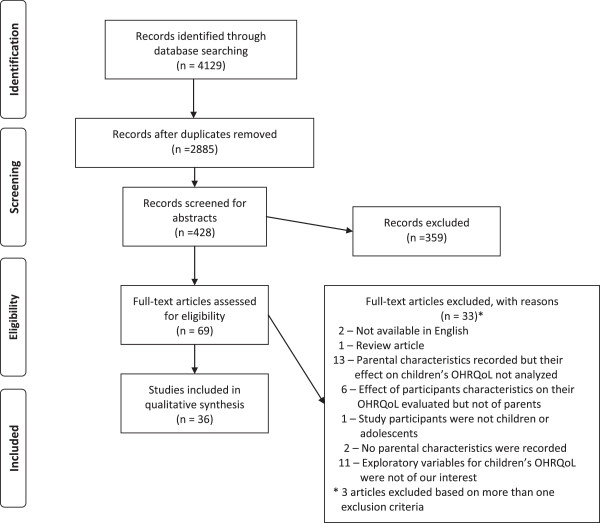
PRISMA flowchart depicting the flow of information through the different phases.

Figure 1 illustrates the details of both the selected and excluded studies. The database search retrieved a total of 5646 titles (2,627 from PubMed, 829 from Medline via OVID, 673 from CINAHL and 1,517 from the COCHRANE). After removing duplicates, 4405 titles remained, and 428 titles were considered for abstract screening. After excluding a further 359 articles based on their abstract, 69 [4,9,14,15,21-85] articles were considered for full-text review of which 36 met the inclusion criteria. For the articles excluded, two were in Portuguese [84,85], one was a review [53] thirteen did not analyse the effect of recorded parental characteristics on children’s OHRQoL [58,61,62,64-66,69,72,74,76-78], [81], six evaluated the effect of socio-demographic characteristics of participants’ on OHRQoL but not of their parents [67,68,73,75,80,82], one was not conducted on children or adolescents [70], two did not collect data on parental characteristics [60,71], eleven analysed the influence of exploratory variables on children’s OHRQoL that were not directly related to parents [9,54-57,59,63,73,74,78,79]. Three articles were excluded based on more than one exclusion criteria [73,74,78].

### Overview of the included studies

#### Year of publication

Approximately one-third of the studies considered for inclusion [4,25,26,28,32,33,38,43], [47,48,50] were published in 2013, while eight [14,21,27,29,31,34,35,83] and six papers [15,22,30,40,44,52] were published in 2012 and 2011 respectively. Aside from one paper published in 2005 [49], there were no papers that pre-dated 2007.

#### Study setting

Of the 36 articles which met the inclusion criteria nearly half (n = 16) were conducted in Brazil, followed by two each from Thailand [34,35], New Zealand [29,50] and Tanzania [39,41]. One study from Thailand [34] was conducted on both 12- and 15-year-old children, with a separate data set presented for both age groups; hence the data from this study appears in both Additional file 1: Table S1 and Table 2. There was one article each from the United States [27], Canada [36], France [49], Hong Kong [52], Malaysia [23], India [15], United Kingdom [32], Saudi Arabia [43], Syria [26], Greece [44], Norway [31], Chile [37], Sudan [42] and Argentina [83].

**Table 2:** Background and study characteristics of studies conducted in adolescents as well as children aged 10-21 years old

**Study design**	**Study sample characteristics**	**Age of the sample**	**Sample size (response rate)**	**OHRQOL instrument and method of administration**	**Parental characteristics studied**	**Significant parental characteristics in unadjusted analysis**	**Significant parental characteristics in adjusted analysis**	**Insignificant parental characteristics**	**Quality**	**Reference**
CS	Public school children in grade 6 of Kilwa district, Tanzania	10-19	1780 (72.6%)	Child-OIDP (Kiswahili version) by interviewing subjects	Mother’s education	Family wealth index on eating and leaning	Family wealth index on eating and speaking	Mother’s education	Moderate	[39]
					Father’s education			Father’s education		
					Family wealth index					
CS	Sub-sample of the sixth Thailand national oral health survey	15	811 (93.1%)	OIDP by interviewing children	Daily pocket money	None	None	Daily pocket money	Moderate	[34]
CS	Secondary school children, Sao Paulo, Brazil	15-16	1060 (48.1%)	OIDP attributed to malocclusion by interviewing the subjects	Socio-economic status (a composite measure recorded based on participation of the head of household in the production or distribution processes)	None	Not conducted	Socio-economic status	Weak	[24]
CS	School children of metropolitan/non-metropolitan and urban/rural areas of Greece	15-18	515	OHIP-14 questionnaire by children in face to face interviews	Parental education	None	Not done	Parental education	Weak	[44]
					Parental Occupation			Parental Occupation		
CS	High school children of the province of Santiago, Chile	12-21	9155 (99.9%)	Modified OHIP-Sp questionnaire by children	Household size	Household size	Household size	None	Moderate	[37]
					Housing status	Housing status	Housing status			
					Number of cars owned by the family	Number of cars owned by the family	Number of cars owned by the family			
					Paternal income	Paternal income	Paternal income			
					Level of mother’s education	Mother’s education	Mother’s education			
CS	Secondary school students of Arusha, Northern Tanzania	12-21	2412 (80.7%)	Child-OIDP (Kiswahili version) questionnaire by subjects	Father’s education	Mother’s education	Family SES	Household wealth index	Moderate	[41]
					Mother’s education	Father’s education	Parents affording dental care			
					Family socio-economic status (perceived affluence of household)	Parents affording dental care				
					Household wealth index (based on durable household assets indicative of family wealth)	Family SES				
					Parents affording dental care					

CS - Cross-sectional; OIDP - Oral Impacts on Daily Performance; OHIP - Oral Health Impact Profile.

#### Age of the study population

Nine studies were conducted on pre-school children (Table 3), while 22 and 6 studies had a study population aged in the range of 10–15 (Additional file 1: Table S1) and 10–21 (Table 2) years respectively.

**Table 3:** Characteristics of the study population and principal results from OHRQoL studies in preschool children (studies involving children aged 6 have also been included)

**Study design**	**Study sample**	**Age of the sample**	**Sample size n (response rate)**	**OHRQOL instrument and method of administration**	**Parental characteristics studied**	**Significant parental characteristics in unadjusted analysis**	**Significant parental characteristics in adjusted analysis**	**Insignificant parental characteristics**	**Quality**	**Reference**
CS	Preschool children who sought dental care during the screening program at University of Sao Paulo, Brazil	2-5	260 (85.2%)	ECOHIS questionnaire by one of the parent	Number of siblings	Household crowding	Family income	Number of siblings	Moderate	[22]
					Marital status of parents	Mother’s work activity		Marital status of parents		
					Household crowding	Family income		House property		
					House property			Mother’s age		
					Family income			Father’s age		
					Mothers’ age			Mother’s education		
					Father’s age			Father’s education		
					Mother’s education			Father’s work activity		
					Father’s education					
					Father’s work activity away from home					
					Mother’s work activity away from home					
Prospective cohort	Interview data from Carolina oral health literacy project	3-5	203	ECOHIS questionnaire by caregivers	Caregiver’s oral health literacy	Caregiver’s age (inversely related to ECOHIS)	Not conducted	None	Moderate	[27]
					Caregiver's age	Caregiver’s education (inversely related to ECOHIS)				
					Caregiver’s education	Caregiver’s oral health literacy (weakly correlated with ECOHIS)				
						(no p values provided for the relationship between any of the variables and children’s OHRQoL)				
CS	Children and mothers of city of Pelotas, Brazil	2-5	608 (88.7%)	ECOHIS questionnaire by mothers	Maternal dental anxiety	Maternal dental anxiety for total ECOHIS score, child’s function and parent distress domain	Maternal dental anxiety for parent distress domain	Family income	Moderate	[30]
					Family income	Mother’s education	Mother’s education for total ECOHIS score			
					Mother’s education	Mother’s use of dental services	Mother’s use of dental service for child’s function domain and total ECOHIS			
					Mother’s use of dental services					
CS	Public prescool/nurseries children of Canoas, Brazil	2-5	1245 (90.2%)	ECOHIS questionnaire by caregivers	Mother’s age	None	None	Mother’s age	Moderate	[33]
					Family structure (nuclear/non-nuclear)			Family structure		
					Mother’s education			Mother’s education		
					Family income			Family income		
CS	Children of Diamentina, Brazil	2-5	638 (98.1%)	ECOHIS by interviewing any of the parent	Marital status of parents	None	Mother’s age	Marital status of parents	Moderate	[38]
					Household crowding			Household crowding		
					Mother’s age			Number of siblings		
					Number of siblings			Mother’s education		
					Mother’s education			Father’s education		
					Father’s education			Family income		
					Family income					
CS	Pre-school children of city of Belo Horizontem, Brazil.	5	1412 (96.3%)	ECOHIS questionnaire by caregivers	Child’s position (single child/others)	Child’s position	*For child impact section (CIS) of ECOHIS*	*For CIS of ECOHIS*	Moderate	[48]
					Caregiver’s age	Caregiver’s age	Child’s position	Caregiver’s education		
					Caregiver’s education	Caregiver’s education	Caregivers age	Caregiver’s relationship to the child		
					Caregiver’s relationship to the child (Mother/others)	Social vulnerability index	Household income	Social Vulnerability Index		
					Household income	Household income	*For Family impact section (FIS)of ECOHIS*	*For FIS of ECOHIS*		
					Social Vulnerability Index (a measure for socioeconomic status based on the residence)		Caregivers age	Child’s position		
							Household income	Caregiver’s education		
								Caregiver’s relationship to the child		
								Social Vulnerability Index		
CS	Chinese preschool children in Hong Kong	3-5	1296 (96.5%)	ECOHIS questionnaire by parents or caregivers	Relationship of the primary caregiver to the child (Mother /other family member)	Caregiver’s education for only symptom domain of ECOHIS	Household income on family impact section of ECOHIS	Relationship of caregiver to the child on overall ECOHIS, FIS and CIS.	Moderate	[52]
					Caregiver’s education	Income level for only symptom domain of ECOHIS		Caregiver’s education for other domains of the ECOHIS except symptoms, overall ECOHIS, FIS and CIS		
					Household income					
								Income level for other domains of the ECOHIS except symptoms, overall ECOHIS, FIS and CIS		
CS	Preschool children of Patumwan district, Bangkok, Thailand	5-6	503 (100% )	Modified Child-OIDP questionnaire by subjects	Family structure (Living with parents/Living with single parents or others)	Hometown of the parents	Occupation of the head of the household	Family structure	Moderate	[35]
					Hometown of parents (Both were from Bangkok/either was from Bangkok/both were from other places)	Occupation of the head of the household				
					Occupation of the head of the household					
CS	Kindergarten children of Buenos Aires metropolitan area, Argentina	Not provided	95 (69.8%)	ECOHIS questionnaire by parents	Family SES or poverty related factors based on parent’s/caregiver’s education, family work conditions and oral health care coverage	SES or poverty related factors on family impact section of ECOHIS	Not conducted	None	Poor	[83]

CS - Cross-sectional; ECOHIS - Early Childhood Oral Health Impact Scale.

#### OHRQoL instruments used

The Early Childhood Oral Health Impact Scale (ECOHIS) was the OHRQoL instrument of choice in preschool children, except for one study [35]. Child Perceptions Questionnaire (CPQ_11-14_) was the most widely used OHRQoL instrument in studies conducted on children and adolescents with fourteen papers reporting its use. Child-OIDP was used in six studies [25,34,39,41,42,49], two of them being the validation studies [42,49]. Parental-Caregivers Perceptions Questionnaire (P-CPQ) and Family Impact Scale (FIS) components of COHQoL without CPQ_11-14_ were used by two studies [21,43]. Three studies used OIDP with study populations aged 12 [45], 15 [34] and 15–16 years [24]. Oral Health Impact Profile (OHIP) was used in three studies, one with a study population aged 12–15 years [28], and the other two with adolescents in the age range of 15–18 [44] and 12–21 [37] years.

#### Quality of the study

Only three articles scored a global rating of “strong” based on EPHPP criteria. Most of the studies were either “moderate” (22 articles) or “weak” (11 articles).

### Socio-economic status (SES)

A broad range of SES indicators were used in different studies; family income, parents’ occupation, parents’ education, family economic status, deprivation status and household wealth index. Seven studies reported of using a single composite scale for SES assessment [24,26,28,32,42,45,83], of which two [42,83] observed poor OHRQoL in children belonging to high SES and one [26] reported of children belonging to low SES having poor OHRQoL. Area-based deprivation was used in three studies [29,48,50], of which one study on intermediate school children of Dunedin observed that those belonging to high deprivation had poorest OHRQoL compared to those in the low and medium categories of deprivation [29], but its effect was not observed in adjusted analysis.

#### Family income

Apart from four articles [15,27,35,44], family income or other indicators of family economy were recorded in all the included articles. Among the sixteen articles that evaluated the influence of total family income on children’s OHRQoL, twelve papers found a significant association [4,14,21-23,36,43,46-48,51,52] with better family income predicting better OHRQoL in children. Although all the studies reported of children from families with high income having better OHRQoL, there were a few discrepancies between the studies; income was significant only in unadjusted analysis [23], effect of family income was limited to overall CPQ score and its two domains [46], family income had significant effect only on symptoms domain and family impact section of ECOHIS [52], family income was significant predictor of FIS of COHRQoL but not P-CPQ [43], income significantly related to overall CPQ_11-14_ and all its domains except for functional limitations [51]. A cohort study that estimated the association of oral health impacts in 12-year-old Brazilian adolescents with life course socio-economic variables considered family income at birth as one of the predictors, but its effect was not presented in the results [45].

#### Other family income indicators

More than half of the thirteen studies that analysed the effect of proxy family income indicators on children’s OHRQoL found them to be significantly related to the outcome. The proxy measures of family income or wealth used in few of the articles were house ownership [40] (only associated with oral symptoms domain of CPQ_11-14_); family wealth index based on durable household assets (related to impairment of few functions) [39]; and family health insurance (was not significantly related to children’s OHRQoL) [49]. The perceived affluence of household, household wealth index based on durable household assets, and parent’s affordability for dental care were proxy measures in a study, and found both household wealth index and parent’s affordability to dental care to be significant predictors of children’s OHRQoL [41]. A study on a representative sample of 12- and 15-year-old children in Thailand found that the prevalence of oral health impacts on QoL in 12-year-old children was greater in those children who receive daily pocket money of 0-20 baht compared to those who receive more than 20 baht per day [34]; this was significant only in unadjusted analysis.

Eight studies used family income along with other proxy measures such as self-perceived family economy [31], receipt of governmental income support and family dental insurance [36], financial government support [25], and social vulnerability index [48], which were found to have a significant effect on children’s OHRQoL in unadjusted analysis. Conversely, house property [21] and house ownership [14] were not found to be significant, but one study on 12-year-old school children of Juiz de For, Brazil, reported that children whose parents owned a house had better OHRQoL than those who do not own a house [4] only in bivariate analysis. A study on Chilean adolescents found number of cars owned and monthly paternal income to be significantly associated with OHRQoL, while house ownership was insignificant [37].

#### Parent’s occupation

Six studies [22,35,44-46,49] analysed the effect of a parent’s occupation on children’s OHRQoL. Of the four studies that observed a significant association, three found associations when the effect of confounders was not controlled in statistical analysis. Preschool children whose household heads were unskilled or economically inactive had a higher likelihood of having high-level oral impacts than those whose household heads had skilled occupations [35], while studies from France [49] and Greece [44] reported the parent’s professional activity and parental occupation, respectively, to be insignificant. The influence of occupation of both the parents on their children’s OHRQoL was studied in an article from Brazil, but the father’s occupation was singularly significant in unadjusted analysis, with children of unemployed fathers being at greater risk of poor OHRQoL than those who had employed fathers [46]. In a study that assessed the effect of parents’ work activity found fathers’ work activity away from home to be insignificant while mothers’ work activity was significantly related to total ECOHIS scores which was not observed after statistical adjustment [22]. Occupational position of the head of the family [24] and occupation of both the parents [26] were used as components of composite SES scale in two studies. Maternal employment status at age 6 months was a significant predictor for children’s OHRQoL at 12 years of age, with children of employed mothers reporting poorer OHRQoL than those who were not working [45].

#### Parent’s education

Twenty-two studies [4,14,21-23,25,30,31,33,36-39], [41,43-48,51,52] assessed the effect of educational level of parents on children’s OHRQoL.

The education level of both parents was found not to be related to children’s OHRQoL in three studies [22,38,39]. Parental education was recorded in two studies (with no clarity if the term ‘parental’ implied mother or father) that found no relationship between parental education and children’s OHRQoL [23,44]. Two studies that evaluated the effect of mother’s education on children’s OHRQoL found it to be insignificant [33,47].

The remaining fifteen studies found mixed results, with few observing significant effects of both parents’ or either of the parent’s education on children’s OHRQoL, and the remaining reporting parent’s education to influence only a few domains of the OHRQoL. However, in all fifteen studies, a higher level of parental education was associated with better OHRQoL in children.

Three studies reported that higher educational level of the mother and father predicted better OHRQoL in children but only the mother’s education was significantly related to OHRQoL after adjustment [4,14,46]. Father’s education was significantly related to children’s OHRQoL among cerebral palsy children in Brazil but it did not maintain its significance after statistical adjustment [21], while another study observed the father’s years of schooling to be significantly related to children’s perceptions of QoL after the effect of other confounders was adjusted in statistical analysis [25]. In a study that was conducted on adolescents in Tanzania, the education level of both the mother and father were significant predictors for better OHRQoL in children in unadjusted analysis alone [41].

Two studies [43,51] observed the mother’s, but not the father’s, education to be significantly related to OHRQoL scores. In four studies, the mother’s and caregiver’s [31,36] education had a significant association with OHRQoL scores in unadjusted analysis [48,52], but in three other studies, higher level of the mother’s education significantly predicted better QoL perceptions in children both in adjusted as well as unadjusted analysis [30,37,45].

Parental education was one of the components of SES scale in five studies [26,28,42,45,83] that evaluated SES’s influence on children’s OHRQoL. In two of these studies [28,45], only the education of the head of the household was integral of SES scale.

### Parent’s demographics

#### Age of the parents

Seven articles [22,25,27,33,38,43,48] considered the effect of parent’s age on children’s OHRQoL. Two articles [22,33] found no significant relationship between parent’s age and children’s OHRQoL. Four studies reported the mother’s [25,38] or caregiver’s [27,48] age to be inversely related to children’s OHRQoL. In one study, only mother’s age was significantly related to both P-CPQ and FIS components of COHRQoL, but the direction of the relationship was not reported in the results [43].

#### Location of origin of the parents

Two studies were identified that recorded information on the parents’ place of origin in relation to the study location [35,49] and found this to be significantly related to children’s OHRQoL. A study conducted on preschool children in Bangkok observed that children of parents whose hometown was not Bangkok were at a greater risk of having a high level of oral impacts than those children of whom either one or both parents were from Bangkok. However, this effect of the parents’ hometown was not evident in an adjusted statistical analysis [35]. Similarly, a study from France observed higher Child-OIDP scores in children whose mothers were immigrants [49], while a father’s place of birth was not significantly related to child-OIDP scores.

#### Marital status of the parents

Two studies [22,38] found no difference between the OHRQoL scores between the children with married and those with unmarried parents.

### Home environment

#### Relationship of the caregiver to the child

Two studies [48,52] found no difference in OHRQoL scores between the preschool children who were taken care of by their mothers and those children whose caregivers were other family members. On the contrary, a study on children with AIDS [40] reported that children whose mother was not their caregiver scored lower on the social wellbeing subscale of CPQ_11-14_ than those children who were cared for by their mothers.

#### Family structure

Seven studies [4,14,15,33,35,36,47] reported on the effect of family structure on children’s OHRQoL which was found to be significant in five studies. The definition of family structure differed between the studies. Two studies [33,47] classified family structure as either “nuclear” and “non-nuclear”, while three studies categorised family structure as “living with both the parents” and “living with single parents or others”. Three studies reported better OHRQoL scores in those children living with their biological parents [4,14] and in nuclear families [47] than their comparative counterparts. A comparison of the perception of OHRQoL between children living with parents and those with no parents revealed better OHRQoL scores in the former [15]. Poor OHRQoL was found in children living in one-adult households than those children from multi-adult households [36].

#### Crowding

Eight studies [4,14,21,22,25,37,38,40] evaluated the effect of household crowding on perceptions of the impact of oral health on quality of life in children. All the studies recorded number of people per room as a measure of house crowding, except for one [37] that observed children in households, with more than three persons reporting poorer OHRQoL than those children with household size of 1–3 persons. Three studies [25,38,40] found household crowding to be an insignificant variable, while four studies [4,14,21,22] found it to be a significant predictor of children’s OHRQoL only when statistical adjustment for confounders was not done.

#### Number of siblings

Eight articles [4,14,21,22,36,38,48,49] studied the relationship of number of siblings or children in the family with OHRQoL. Three studies [21,22,38] did not find any influence of the number of siblings on children’s OHRQoL, while an equal number of studies [4,14,49] observed that the perception of OHRQoL deteriorated as the number of children in the family increased. Two studies [4,48] found that the impact of OHRQoL was poorer in children who had siblings than those who did not have any siblings.

#### Cigarette, alcohol and drug use

One study [14] analysed the effect of cigarette, alcohol and drug use in the family on children’s OHRQoL, and reported poor OHRQoL in children of those families using these products.

### Parental oral health literacy, behaviour and dental anxiety

A study [27] that aimed to find the relationship of caregiver’s oral health literacy with preschool’s OHRQoL found these variables to be weakly correlated. In another study, mothers with moderate or high dental anxiety reported a higher total ECOHIS score but in adjusted analysis, maternal dental anxiety was only associated with the parent distress domain [30]. In the same study, mothers who visited a dentist more frequently reported higher ECOHIS scores [30].

## Discussion

In evaluating oral health, interferences in physical, psychological and social functioning are important [86], as the traditional epidemiologic clinical indicators do not provide an insight into individual’s abilities in performing their roles and activities [87]. Most of the currently available OHRQoL instruments have succeeded in measuring the impact of oral health on physical, functional, social and emotional wellbeing of an individual. Children like adults are also prone for various oral disorders, all of which can likely compromise functioning, well-being and QoL [17]. But the concept of OHRQoL in children has increased dramatically only in recent years. A systematic literature review reported that the number of articles published on child OHRQoL between 2000 and 2006 was three times higher than between 1995 and 1999 [17].

The Wilson and Cleary model of health-related QoL demonstrates that individual perceptions of QoL are influenced by several individual, environmental characteristics and also non-medical factors [16]. However, evaluating the determinants of OHRQoL in children seems to be a new concept as there were no studies older than 2005 in spite of certain OHRQoL instruments being introduced between 2002 and 2005. Admittedly, the OHRQoL instruments for preschool children, such as ECOHIS [10] and SOHO-5 [11], were developed in 2007 and 2012 respectively. The latter instrument is a self-reported OHRQoL measure for 5-year-old children. While the ECOHIS questionnaire was widely used in preschool children, there were no studies that have used SOHO-5 [11], which might be due to its recent development. The CPQ_11-14_ was the most widely used self-reported OHRQoL instrument in studies that were conducted on children and adolescents, and it is found to be valid as well as reliable in many cultural settings [88]. Although the literature on the determinants of children’s OHRQoL is abundant, it is unequally represented, with more than half of the studies conducted in Brazil.

This review indicates that the findings on the correlates of OHRQoL from studies are varied and non-uniform, with different measures being considered by different authors. Moreover, not all studies included in the review aimed to test the association between the parental attributes and children’s OHRQoL. Findings from both the adjusted and unadjusted analysis were tabulated separately for each study. In a few studies, the significant effect of exposure on the outcome that was observed in unadjusted analysis was not observed in multivariate analyses after adjusting for the effect of confounders. The importance of statistical adjustment becomes more pronounced in cross-sectional studies, and especially in those studies that aim to ascertain the influence of many interrelated exposures on an outcome.

Most of the studies were of moderate quality and only three were strong. This is because of the quality assessment criteria used, which rates only those whose study designs are experimental or longitudinal in nature as good. However, experimental or longitudinal study designs are rarely used in OHRQoL studies of our interest. Furthermore, a few studies that were of moderate quality were rated as weak in ‘selection bias’ component of EPHPP as they did not report response rates in the articles. There were four prospective studies [23,27,28,45] one of which was conducted with an objective to evaluate the effect of orthodontic treatment on OHRQoL [28]. Due to the static nature of the exposure data (i.e., socio-economic and home environment characteristics), most of the studies were of cross-sectional design. However, it would be interesting to observe the dynamic effect of these exposure characteristics along the life course on children’s OHRQoL, which was done in one of the studies [45] that assessed the influence of early life social conditions on children’s OHRQoL.

The composite measure of SES or area-based deprivation failed to show its effect on children’s OHRQoL in most of the studies. However, family income or family economy indicators and parental education levels were found to be significant predictors of children’s OHRQoL. Nevertheless, their effect was not observed after adjusted analysis in a few of the studies. Further, the influence of family economy or parental education was associated with only few dimensions of children’s OHRQoL. This discrepancy in results between the studies is due to the statistical methods adopted, i.e., a few studies analysed the effect of family income or parental education on overall OHRQoL score, while the others analysed the effect of these socio-economic variables on overall OHRQoL, as well as its dimensions. In addition, some studies performed statistical adjustment for the effect of confounders when analysing the influence of parental characteristics on children’s OHRQoL and few have not made any attempt to do so. As anticipated, family economy and parental education were directly proportional to children’s OHRQoL in all the studies that have found significant associations. Children of parents with high educational level and family income were more likely to have better OHRQoL. Low educational level may lead to reduced income [13] and lower income is related to material deprivation [46]. Children from poor families have limited access to health care and preventive interventions which might lead to a poor quality of life [14]. None of the studies observed parents’ occupation to be significantly associated with children’s OHRQoL. Based on the findings from a few studies, it can be conceptually summarised that a mother’s work activity is a significant predictor during the early childhood while father’s occupation is significant during late childhood.

Mothers’ or caregivers’ age significantly predicted better OHRQoL in children, which might be due to younger mothers feeling less secure in caring for their child [38]. Moreover, children of parents who are not native to the study location were found to be more prone to poor OHRQoL than those children whose parents are native to the area. This might be due to the indirect influence of SES, as migrants tend to have a lower SES than others. The marital status of the parents failed to influence children’s OHRQoL. Mother or other family members being the caregiver of the family did not influence children’s OHRQoL, except in one study on children with AIDS [40]. It might be because of the additional care needed by these children than others as they are more prone to poor oral health. It is evident from the studies reviewed that children living with biological parents and those with nuclear families have better OHRQoL. More than half the studies that evaluated the relationship of crowding found it to be significantly associated with children’s OHRQoL, but only in unadjusted analysis. Household crowding is a proxy indicator of SES [89], and thus its association with children’s OHRQoL might have been masked by SES in adjusted analysis. Single children reported lesser impact of oral health on quality of life than those who have siblings, while the effect of the number of siblings a child has on their OHRQoL is inconclusive from the results of the reviewed studies. Other factors that significantly influence children’s OHRQoL comprise familial use of deleterious substances, maternal dental anxiety and dental services usage.

This is first study that has systematically reviewed the literature on the effect of parental socio-economic and home environment characteristics on children’s OHRQoL. A systematic review [53] has been published recently that evaluated the effect of socio-economic characteristics on OHRQoL, which also included studies on children. In order to avoid exclusion of potential articles that had keywords other than those we have used, a broader term “Oral Health Related Quality of Life” was used to search “all fields”. We have not included other studies with the predictors “ethnicity”, “urbanisation”, “school type”, “dental fear” and “dental visits” as these are not directly related to either socio-economic or home environment characteristics. One of the limitations of the present review is the lack of quantitative data presentation by meta-analysis. Meta-analysis was not possible due to extremely heterogeneous data from the studies included, with categorisation of both the outcomes and exploratory variables differing between the studies.

## Conclusions

Accurate conclusions from the studies reviewed are not possible due to the difference in the study population, methods used and statistical tests performed. In general, children from families with high income, parental education and family economy had better OHRQoL. Mothers’ age and home environment characteristics, such as family structure, household crowding and presence of siblings were significantly related to the outcome. Although the association of children’s OHRQoL and variables like location of origin of parents in relation to study location, deleterious habits in the family, mother’s dental anxiety and use of dental services were significant, the evidence is not strong enough as the data supporting their relationship with the outcome is only from one study. Lastly, the conclusions from the current review cannot be generalised to the whole population as the studies reviewed were not representative from the whole world, and nearly half of the articles were Brazil-based studies.

## Competing interests

The authors declare that they have no competing interests.

## Authors’ contributions

SK, JK and RL participated in designing and developing the literature search protocol for the review. SK conducted the literature search, extracted and interpreted the data from relevant articles. JK and RL contributed in scrutinising the data extraction. All the authors contributed in drafting and approval of the final manuscript.

## Supplementary Material

Additional file 1: Table S1. Overview of the studies on children between the ages 10 – 15 years. Click here for file

## References

[B1] The WHOQOL Group The world Health Organization Quality of life assessment (WHOQOL): position paper from the World Health Organization Soc Sci Med 1995 41 1403 1409 8560308 10.1016/0277-9536(95)00112-k

[B2] Llewellyn CD Warnakulasuriya S The impact of stomatological disease on oral health-related quality of life Eur J Oral Sci 2003 111 297 304 10.1034/j.1600-0722.2003.00057.x 12887394

[B3] McGrath C Broder H Wilson-Genderson M Assessing the impact of oral health on the life quality of children: implications for research and practice Community Dentistry Oral Epidemiol 2004 32 81 85 10.1111/j.1600-0528.2004.00149.x 15061856

[B4] de Paula JS LI de Almeida AB Ambrosano GM Mialhe F The impact of socioenvironmental characteristics on domains of oral health-related quality of life in Brazilian schoolchildren BMC Oral Health 2013 28 13:10 10.1186/1472-6831-13-10 PMC3573924 23356655

[B5] Barbosa TS Gaviao MB Oral health-related quality of life in children: part I: how well do children know themselves? A systematic review Int J Dent Hyg 2008 6 93 99 10.1111/j.1601-5037.2007.00276.x 18412720

[B6] Jokovic A Locker D Stephens M Kenny D Tompson B Guyatt G Validity and reliability of a questionnaire for measuring child oral-health-related quality of life J Dent Res 2002 81 459 463 10.1177/154405910208100705 12161456

[B7] Jokovic A Locker D Tompson B Guyatt G Questionnaire for measuring oral health-related quality of life in eight- to ten-year-old children Pediatr Dent 2004 26 512 518 15646914

[B8] Gherunpong STG Sheiham A Developing and evaluating an oral health-related quality of life index for children; the CHILD-OIDP Community Dent Health 2004 21 161 169 15228206

[B9] Broder HL Wilson-Genderson M Reliability and convergent and discriminant validity of the Child Oral Health Impact Profile (COHIP Child’s version) Community Dentistry Oral Epidemiol 2007 35 Suppl 1 20 31 10.1111/j.1600-0528.2007.0002.x 17615047

[B10] Pahel BT Rozier RG Slade GD Parental perceptions of children’s oral health: the early childhood oral health impact scale (ECOHIS) Health Qual Life Outcomes 2007 5 6 10.1186/1477-7525-5-6 17263880 PMC1802739

[B11] Tsakos G Blair YI Yusuf H Wright W Watt RG Macpherson LM Developing a new self-reported scale of oral health outcomes for 5-year-old children (SOHO-5) Health Qual Life Outcomes 2012 10 62 10.1186/1477-7525-10-62 22676710 PMC3413607

[B12] Santhosh KJT Prabu D Suhas K Socio-behavioral variables effecting oral hygiene and periodontal status of 12 year-old schoolchildren of Udaipur district Odontostomatol Trop 2013 36 27 33 23781683

[B13] Sanders AE Spencer AJ Childhood circumstances, psychosocial factors and the social impact of adult oral health Community Dent Oral Epidemiol 2005 33 370 377 10.1111/j.1600-0528.2005.00237.x 16128797

[B14] Paula JS Leite IC Almeida AB Ambrosano GM Pereira AC Mialhe FL The influence of oral health conditions, socioeconomic status and home environment factors on schoolchildren’s self-perception of quality of life Health Qual Life Outcomes 2012 10 6 10.1186/1477-7525-10-6 22244092 PMC3285522

[B15] Kumar SGA Tadakamadla J Tibdewal H Duraiswamy P Kulkarni S Oral health related quality of life among children with parents and those with no parents Community Dent Health 2011 28 227 231 21916359

[B16] Wilson IB Cleary PD Linking clinical variables with health-related quality of life: a conceptual model of patient outcomes JAMA 1995 273 59 65 10.1001/jama.1995.03520250075037 7996652

[B17] Barbosa TS Gaviao MB Oral health-related quality of life in children: part II: effects of clinical oral health status: a systematic review Int J Dent Hyg 2008 6 100 107 10.1111/j.1601-5037.2008.00293.x 18412721

[B18] Moher D Liberati A Tetzlaff J Altman DG Preferred reporting items for systematic reviews and meta-analyses: the PRISMA statement BMJ 2009 339 b2535 10.1136/bmj.b2535 19622551 PMC2714657

[B19] Effective Public Health Practice Project. Quality assessment Tool for Quantitative Studies http://www.ephpp.ca/PDF/Quality%20Assessment%20Tool_2010_2.pdf

[B20] Chillon P Evenson KR Vaughn A Ward DS A systematic review of interventions for promoting active transportation to school Int J Behav Nutr Phys Act 2011 8 10 10.1186/1479-5868-8-10 21320322 PMC3050785

[B21] Abanto J Carvalho TS Bonecker M Ortega AO Ciamponi AL Raggio DP Parental reports of the oral health-related quality of life of children with cerebral palsy BMC Oral Health 2012 12 15 10.1186/1472-6831-12-15 22708973 PMC3500272

[B22] Abanto J Carvalho TS Mendes FM Wanderley MT Bönecker M Raggio DP Impact of oral diseases and disorders on oral health-related quality of life of preschool children Community Dentistry Oral Epidemiol 2011 39 105 114 10.1111/j.1600-0528.2010.00580.x 21029148

[B23] Baker SR Mat A Robinson PG What psychosocial factors influence adolescents’ oral health? J Dent Res 2010 89 1230 1235 10.1177/0022034510376650 20739689

[B24] Bernabe E Tsakos G Messias de Oliveira C Sheiham A Impacts on daily performances attributed to malocclusions using the condition-specific feature of the oral impacts on daily performances index Angle Orthod 2008 78 241 247 10.2319/030307-111.1 18251604

[B25] Carvalho JC Rebelo MA Vettore MV The relationship between oral health education and quality of life in adolescents Int J Paediatr Dent 2013 23 286 296 10.1111/ipd.12006 23113917

[B26] Dak-Albab RJ Dashash MA The influence of socioeconomic status on oral health-related quality of life among Syrian children with cleft lip, or palate, or both Saudi Med J 2013 34 181 186 23396466

[B27] Divaris K Lee JY Baker AD Vann WF Jr Caregivers’ oral health literacy and their young children’s oral health-related quality-of-life Acta Odontol Scand 2012 70 390 397 10.3109/00016357.2011.629627 22150574 PMC3305855

[B28] Feu D Miguel JA Celeste RK Oliveira BH Effect of orthodontic treatment on oral health-related quality of life Angle Orthod 2013 83 892 898 10.2319/100412-781.1 23593976 PMC8744507

[B29] Foster Page LA Thomson WM Ukra A Farella M Factors influencing adolescents’ oral health-related quality of life (OHRQoL) Int J Paediatr Dent 2013 23 415 423 23171387 10.1111/ipd.12011

[B30] Goettems ML Ardenghi TM Romano AR Demarco FF Torriani DD Influence of maternal dental anxiety on oral health-related quality of life of preschool children Qual Life Res 2011 20 951 959 10.1007/s11136-010-9816-0 21181500

[B31] Koposova N Eriksen HM Widstram E Eisemann M Opravin A Koposov R Oral health-related quality of life among 12-year-olds in Northern Norway and North-West Russia Oral Health Dental Manage 2012 11 206 214 23208598

[B32] Kotecha S Turner PJ Dietrich T Dhopatkar A The impact of tooth agenesis on oral health-related quality of life in children J Orthod 2013 40 122 129 10.1179/1465313312Y.0000000035 23794692

[B33] Kramer PF Feldens CA Helena Ferreira S Bervian J Rodrigues PH Peres MA Exploring the impact of oral diseases and disorders on quality of life of preschool children Community Dentistry Oral Epidemiol 2013 41 327 335 10.1111/cdoe.12035 23330729

[B34] Krisdapong S Prasertsom P Rattanarangsima K Sheiham A Tsakos G The impacts of gingivitis and calculus on Thai children’s quality of life J Clin Periodontol 2012 39 834 843 10.1111/j.1600-051X.2012.01907.x 22783901

[B35] Krisdapong S Somkotra T Kueakulpipat W Disparities in early childhood caries and its impact on oral health-related quality of life of preschool children Asia Pac J Public Health 2012 Mar 16. [Epub ahead of print] 10.1177/1010539512438608 22426563

[B36] Locker D Disparities in oral health-related quality of life in a population of Canadian children Community Dentistry Oral Epidemiol 2007 35 348 356 10.1111/j.1600-0528.2006.00323.x 17822483

[B37] López R Baelum V Oral health impact of periodontal diseases in adolescents J Dent Res 2007 86 1105 1109 10.1177/154405910708601116 17959905

[B38] Martins-Júnior PA Vieira-Andrade RG Corrêa-Faria P Oliveira-Ferreira F Marques LS Ramos-Jorge ML Impact of early childhood caries on the oral health-related quality of life of preschool children and their parents Caries Res 2013 47 211 218 10.1159/000345534 23257929

[B39] Mashoto KO Astrøm AN David J Masalu JR Dental pain, oral impacts and perceived need for dental treatment in Tanzanian school students: a cross-sectional study Health Qual Life Outcomes 2009 7 73 73 10.1186/1477-7525-7-73 19643004 PMC2726126

[B40] Massarente DB Domaneschi C Marques HHS Andrade SB Goursand D Antunes JLF Oral health-related quality of life of paediatric patients with AIDS BMC Oral Health 2011 11 7p 10.1186/1472-6831-11-7 21208437 PMC3020164

[B41] Mbawalla HS Masalu JR Astrøm AN Socio-demographic and behavioural correlates of oral hygiene status and oral health related quality of life, the Limpopo-Arusha school health project (LASH): a cross-sectional study BMC Pediatr 2010 10 87 87 10.1186/1471-2431-10-87 21118499 PMC3001697

[B42] Nurelhuda NM Ahmed MF Trovik TA Astrøm AN Evaluation of oral health-related quality of life among Sudanese schoolchildren using Child-OIDP inventory Health Qual Life Outcomes 2010 8 152 152 10.1186/1477-7525-8-152 21182769 PMC3019139

[B43] Pani SC Mubaraki SA Ahmed YT Alturki RY Almahfouz SF Parental perceptions of the oral health-related quality of life of autistic children in Saudi Arabia Spec Care Dentist 2013 33 8 12 10.1111/j.1754-4505.2012.00294.x 23278143

[B44] Papaioannou W Oulis CJ Latsou D Yfantopoulos J Oral health related quality of life of Greek adolescents: a cross-sectional study Eur Arch Paediatr Dent 2011 12 146 150 10.1007/BF03262796 21640059

[B45] Peres KG Peres MA Araujo CL Menezes AM Hallal PC Social and dental status along the life course and oral health impacts in adolescents: a population-based birth cohort Health Qual Life Outcomes 2009 7 95 10.1186/1477-7525-7-95 19930601 PMC2785763

[B46] Piovesan C Antunes JL Guedes RS Ardenghi TM Impact of socioeconomic and clinical factors on child oral health-related quality of life (COHRQoL) Qual Life Res 2010 19 1359 1366 10.1007/s11136-010-9692-7 20571918

[B47] Scapini A Feldens CA Ardenghi TM Kramer PF Malocclusion impacts adolescents’ oral health-related quality of life Angle Orthod 2013 83 512 518 10.2319/062012-509.1 23210545 PMC8763080

[B48] Scarpelli AC Paiva SM Viegas CM Carvalho AC Ferreira FM Pordeus IA Oral health-related quality of life among Brazilian preschool children Community Dentistry Oral Epidemiol 2013 41 336 344 10.1111/cdoe.12022 23253051

[B49] Tubert-Jeannin S Pegon-Machat E Gremeau-Richard C Lecuyer MM Tsakos G Validation of a French version of the Child-OIDP index Eur J Oral Sci 2005 113 355 362 10.1111/j.1600-0722.2005.00230.x 16202021

[B50] Ukra A Thomson WM Farella M Tawse Smith A Beck V Impact of malocclusion on quality of life among New Zealand adolescents N Z Dent J 2013 109 18 23 23923152

[B51] Vargas-Ferreira F Piovesan C Praetzel JR Mendes FM Allison PJ Ardenghi TM Tooth erosion with low severity does not impact child oral health-related quality of life Caries Res 2010 44 531 539 10.1159/000321447 21051891

[B52] Wong HM King NM Oral health-related quality of life in Hong Kong preschool children Caries Res 2011 45 370 376 10.1159/000330231 21822015

[B53] Cohen-Carneiro F Souza-Santos R Rebelo MA Quality of life related to oral health: contribution from social factors Ciencia Saude Coletiva 2011 16 Suppl 1 1007 1015 21503449 10.1590/s1413-81232011000700033

[B54] Acharya STS The effect of early childhood caries on the quality of life of children and their parents Contemp Clin Dent 2011 2 98 101 10.4103/0976-237X.83069 21957384 PMC3180838

[B55] Foster Thomson WM Mohamed AR Traebert J Performance and cross-cultural comparison of the short-form version of the CPQ_11-14_ in New Zealand, Brunei and Brazil Health Qual Life Outcomes 2011 9 40 40 10.1186/1477-7525-9-40 21649928 PMC3130632

[B56] Krisdapong S Prasertsom P Rattanarangsima K Sheiham A Sociodemographic differences in oral health-related quality of life related to dental caries in Thai school children Community Dent Health 2013 30 112 118 23888542

[B57] Luoto A Lahti S Nevanpera T Tolvanen M Locker D Oral-health-related quality of life among children with and without dental fear Int J Paediatr Dent 2009 19 115 120 10.1111/j.1365-263X.2008.00943.x 19250394

[B58] Pani SC Badea L Mirza S Elbaage N Differences in perceptions of early childhood oral health-related quality of life between fathers and mothers in Saudi Arabia Int J Paediatr Dent 2012 22 244 249 10.1111/j.1365-263X.2011.01185.x 22010957

[B59] Yazicioglu I Jones JA Cortes D Rich S Garcia R Hispanic parents’ reading language preference and pediatric oral health-related quality of life J Public Health Dent 2013 73 329 338 10.1111/jphd.12031 23968305

[B60] Zhang M McGrath C Hagg U Who knows more about the impact of malocclusion on children’s quality of life, mothers or fathers? Eur J Orthod 2007 29 180 185 10.1093/ejo/cjl058 17489000

[B61] Alves LS Damé-Teixeira N Susin C Maltz M Association among quality of life, dental caries treatment and intraoral distribution in 12-year-old South Brazilian schoolchildren Community Dentistry Oral Epidemiol 2013 41 22 29 10.1111/j.1600-0528.2012.00707.x 22882480

[B62] Bendo CB Paiva SM Torres CS Oliveira AC Goursand D Pordeus IA Vale MP Association between treated/untreated traumatic dental injuries and impact on quality of life of Brazilian schoolchildren Health Qual Life Outcomes 2010 8 114 10.1186/1477-7525-8-114 20920332 PMC2959098

[B63] Bernabe E Sheiham A de Oliveira CM Impacts on daily performances attributed to malocclusions by British adolescents J Oral Rehabil 2009 36 26 31 10.1111/j.1365-2842.2008.01899.x 18976263

[B64] Biazevic MG Rissotto RR Michel-Crosato E Mendes LA Relationship between oral health and its impact on quality of life among adolescents Pesqui Odontol Bras = Braz Oral Res 2008 22 36 42 10.1590/s1806-83242008000100007 18425243

[B65] Christopherson EA Briskie D Inglehart MR Objective, subjective, and self-assessment of preadolescent orthodontic treatment need–a function of age, gender, and ethnic/racial background? J Public Health Dent 2009 69 9 17 10.1111/j.1752-7325.2008.00089.x 18662255

[B66] Cortes MI Marcenes W Sheiham A Impact of traumatic injuries to the permanent teeth on the oral health-related quality of life in 12-14-year-old children Community Dentistry Oral Epidemiol 2002 30 193 198 10.1034/j.1600-0528.2002.300305.x 12000342

[B67] Crocombe LA Brennan DS Slade GD The influence of dental attendance on change in oral health-related quality of life Community Dentistry Oral Epidemiol 2012 40 53 61 10.1111/j.1600-0528.2011.00634.x 21883354

[B68] Crocombe LA Broadbent JM Thomson WM Brennan DS Poulton R Impact of dental visiting trajectory patterns on clinical oral health and oral health-related quality of life J Public Health Dent 2012 72 36 44 10.1111/j.1752-7325.2011.00281.x 22316176

[B69] Dame-Teixeira N Alves LS Ardenghi TM Susin C Maltz M Traumatic dental injury with treatment needs negatively affects the quality of life of Brazilian schoolchildren Int J Paediatr Dent 2013 23 266 273 10.1111/ipd.12002 23016995

[B70] Fisher MA Gilbert GH Shelton BJ A cohort study found racial differences in dental insurance, utilization, and the effect of care on quality of life J Clin Epidemiol 2004 57 853 857 10.1016/j.jclinepi.2004.01.002 15485738

[B71] Gaynor WN Thomson WM Changes in young children’s OHRQoL after dental treatment under general anaesthesia Int J Paediatr Dent 2012 22 258 264 10.1111/j.1365-263X.2011.01190.x 21999137

[B72] Goettems ML Ardenghi TM Demarco FF Romano AR Torriani DD Children’s use of dental services: influence of maternal dental anxiety, attendance pattern, and perception of children’s quality of life Community Dentistry Oral Epidemiol 2012 40 451 458 10.1111/j.1600-0528.2012.00694.x 22537392

[B73] Hobdell M Tsakos G Sprod A Ladrillo TE Ross MW Gordon N Myburgh N Lalloo R Using an oral health-related quality of life measure in three cultural settings Int Dent J 2009 59 381 388 20162952

[B74] Koposova N Widstrom E Eisemann M Koposov R Eriksen HM Oral health and quality of life in Norwegian and Russian school children: a pilot study Stomatologija 2010 12 10 16 20440091

[B75] McGrath C Bedi R Measuring the impact of oral health in life quality in two national surveys – functionalist versus hermeneutic approaches Community Dentistry Oral Epidemiol 2002 30 254 259 10.1034/j.1600-0528.2002.300403.x 12147167

[B76] Piovesan C Abella C Ardenghi TM Child oral health-related quality of life and socioeconomic factors associated with traumatic dental injuries in schoolchildren Oral Health Prev Dent 2011 9 405 411 22238740

[B77] Roumani T Oulis CJ Papagiannopoulou V Yfantopoulos J Validation of a Greek version of the oral health impact profile (OHIP-14) in adolescents Eur Arch Paediatr Dent 2010 11 247 252 10.1007/BF03262756 20932400

[B78] Traebert J Foster Page LA Thomson WM Locker D Differential item functioning related to ethnicity in an oral health-related quality of life measure Int J Paediatr Dent 2010 20 435 441 10.1111/j.1365-263X.2010.01066.x 20642468

[B79] Vargas-Ferreira F Developmental enamel defects and their impact on child oral health-related quality of life Pesqui Odontol Bras = Braz Oral Res 2011 25 531 537 10.1590/s1806-83242011000600010 22147234

[B80] Williams S Parker E Jamieson L Oral health-related quality of life among rural-dwelling Indigenous Australians Aust Dent J 2010 55 170 176 10.1111/j.1834-7819.2010.01220.x 20604759

[B81] Wong HMJ King NM Rasch validation of the early childhood oral health impact scale Community Dentistry Oral Epidemiol 2011 39 449 457 10.1111/j.1600-0528.2011.00614.x 21504439

[B82] Zanatta FB Ardenghi TM Antoniazzi RP Pinto TM Rosing CK Association between gingival bleeding and gingival enlargement and oral health-related quality of life (OHRQoL) of subjects under fixed orthodontic treatment: a cross-sectional study BMC Oral Health 2012 12 53 10.1186/1472-6831-12-53 23186371 PMC3534331

[B83] Bordoni N Ciaravino O Zambrano O Villena R Beltran-Aguilar E Squassi A Early Childhood Oral Health Impact Scale (ECOHIS): translation and validation in Spanish language Acta Odontol Latinoam 2012 25 270 278 23798073

[B84] Motta LJ Tanizaga NH Guedes CC Santos Mesquita-Ferrari RA Bussadori SK Impact of oral health on quality of life of children from 6 to 10 years [Portuguese] ConScientiae Saude 2011 10 715 722

[B85] Peres KG Latorre Mdo R Peres MA Traebert J Panizzi M Impact of dental caries and dental fluorosis on 12-year-old schoolchildren’s self-perception of appearance and chewing [Portuguese] Cad Saude Publica 2003 19 323 330 10.1590/S0102-311X2003000100037 12700814

[B86] Sheiham A Oral health, general health and quality of life Bull World Health Organ 2005 83 644 16211151 PMC2626333

[B87] Sheiham A Steele JG Marcenes W Tsakos G Finch S Walls AW Prevalence of impacts of dental and oral disorders and their effects on eating among older people; a national survey in Great Britain Community Dent Oral Epidemiol 2001 29 195 203 10.1034/j.1600-0528.2001.290305.x 11409678

[B88] Olivieri A Ferro R Benacchio L Besostri A Stellini E Validity of Italian version of the child perceptions questionnaire (CPQ_11-14_) BMC Oral Health 2013 13 55 10.1186/1472-6831-13-55 24131892 PMC3856540

[B89] Antunes JL Frazao P Narvai PC Bispo CM Pegoretti T Spatial analysis to identify differentials in dental needs by area-based measures Community Dent Oral Epidemiol 2002 30 133 142 10.1034/j.1600-0528.2002.300207.x 12000354

