# Supplementary material for: A systematic review of the impact of parental socio-economic status and home environment characteristics on children’s oral health related quality of life
Source: Health Qual Life Outcomes. 2014 Mar 21;12:41. doi: 10.1186/1477-7525-12-41 (PMC4000002; doi:10.1186/1477-7525-12-41)
Supplement: Additional file 1: Table S1. — Overview of the studies on children between the ages 10 – 15 years. [file 1477-7525-12-41-S1.doc]

**Additional file 1: Table S1** Overview of the studies on children between the ages 10 – 15 years

| Study design | Study sample characteristics | Age of  the sample | Sample size  (response rate) | OHRQOL  instrument and  method of administration | Parental characteristics  studied | Significant  parental characteristics in unadjusted  analysis | Significant parental characteristics in adjusted analysis | Insignificant parental  characteristics | Quality | Reference |
| --- | --- | --- | --- | --- | --- | --- | --- | --- | --- | --- |
| CS | School children of Clermont-Ferrand, France. | 10 | 414 (84%) | Child-OIDP questionnaire by  children in face to face interview (translated  and validated in the same study) | Professional  activity of the father | Place of mothers  birth | Not done | Professional activity of  the father | Weak | [49] |
|  |  |  |  |  | Professional  activity of the  mother | Number of children in the family |  | Professional activity  of the mother |  |  |
|  |  |  |  |  | Place of mother’s birth (France/other country) |  |  | Place of father’s birth |  |  |
|  |  |  |  |  | Place of father’s birth (France/other country) |  |  | Family health insurance |  |  |
|  |  |  |  |  | Number of children in the family |  |  |  |  |  |
|  |  |  |  |  | Family health insurance |  |  |  |  |  |
| CS | School children of Juiz de Fora, Brazil | 12 | 286 | CPQ_11-14_ questionnaire by children | Family structure  (living with biological parents: Yes/no) | Family income | Family income | Presence of siblings | Moderate | [4] |
|  |  |  |  |  | Presence of siblings | Father’s education | Mother’s education |  |  |  |
|  |  |  |  |  | Number of siblings | Mother’s education | Family structure only for domains social and emotional wellbeing. |  |  |  |
|  |  |  |  |  | Household  crowding | Home ownership only with emotional wellbeing domain |  |  |  |  |
|  |  |  |  |  | Family income | Family structure |  |  |  |  |
|  |  |  |  |  | Mother’s education | Number of siblings |  |  |  |  |
|  |  |  |  |  | Father’s education | Household crowding only oral symptoms and social wellbeing domains |  |  |  |  |
|  |  |  |  |  | Home ownership |  |  |  |  |  |
| CS | Schoolchildren of Arkhangelsk, Russia and Tromso, Norway | 12 | 514 from Arkhangelsk (87%) and 124 from Norway (47%) | CPQ_11-14_  questionnaire by children | Mother’s education | Mother’s education | Not done | Social support | Weak | [31] |
|  |  |  |  |  | Family economy | Family economy |  |  |  |  |
|  |  |  |  |  | Social support |  |  |  |  |  |
| CS | School children of Khartoum, Sudan | 12 | 1109 (99%) | Child-OIDP (Arabic-validated in the same study) by interviewing subjects | Socio-economic  status (SES) assessed based on 9 dichotomous  indicators of SES | SES | SES only in public school attendees but not in private school attendees | None | Moderate | [42] |
| CS | School children of the city of Juiz de Fora, Brazil | 12 | 515 | CPQ_11-14_ questionnaire by children | Family structure  (living with both biological parents: Yes/no) | Family structure | Family income | House ownership | Weak | [14] |
|  |  |  |  |  | Number of siblings | Number of siblings | Mother’s education |  |  |  |
|  |  |  |  |  | Use of cigarettes, alcohol and  drug in the family | Use of cigarettes, alcohol and drug in the family | Number of siblings |  |  |  |
|  |  |  |  |  | Household overcrowding | Household overcrowding | Household crowding |  |  |  |
|  |  |  |  |  | Family income | Family income |  |  |  |  |
|  |  |  |  |  | Mothers education | Mother’s education |  |  |  |  |
|  |  |  |  |  | Father’s education | Father’s education |  |  |  |  |
|  |  |  |  |  | House ownership |  |  |  |  |  |
| CS nested in Cohort | Birth Cohort of children born in 1993 in Pelotas, Brazil at age 12 | 12 | 339 (94.4%) | OIDP (modified) questionnaire by children | Family income  at child birth | Maternal schooling | Maternal schooling | Family socio-economic status | Strong | [45] |
|  |  |  |  |  | Maternal  schooling at child aged 6 months | Maternal  employment status | Maternal employment status |  |  |  |
|  |  |  |  |  | Maternal employment status at child aged 6 months |  |  |  |  |  |
|  |  |  |  |  | Family economic status (based on Brazil Criterion for Economic Classification) at child aged 12 years |  |  |  |  |  |
| CS | Public school children of Santa Maria, RS, Brazil | 12 | 792 (90%) | CPQ_11-14_  questionnaire by children | Fathers’ education | Mother’s education | Household income for overall CPQ score, Oral symptoms and emotional wellbeing | Mother’s occupation | Moderate | [46] |
|  |  |  |  |  | Mother’s education | Father’s education | Mother’s education for overall CPQ score, Functional limitations, emotional and social wellbeing domains | Father’s occupation for oral symptoms, functional limitations and social wellbeing domains |  |  |
|  |  |  |  |  | Father’s Occupation | Household income |  |  |  |  |
|  |  |  |  |  | Mother’s Occupation | Father’s occupation only for overall CPQ score and emotional wellbeing |  |  |  |  |
|  |  |  |  |  | Household income |  |  |  |  |  |
| CS | Sub-sample of the sixth Thailand national oral health survey | 12 | 1,063(96.6%) | Child-OIDP by interviewing children | Daily pocket money | Daily pocket money | None | None | Moderate | [34] |
| Prospective | School children of Banting district, Selangor, Malaysia. | 12-13 | 439 (96.9%) | CPQ_11-14_  questionnaire by children | Family income | Family income | None | Parental education | Strong | [23] |
|  |  |  |  |  | Parental education |  |  |  |  |  |
| CS | School children of Udaipur, India | 12-15 | 536 (98.3%) | CPQ_11-14_ by  interviewing the children | Living with  parents/no parents | Living with  parents/no parents | Living with parents/no parents | None | Moderate | [15] |
| CS | Children with Cerebral palsy attending dental clinics of University of Sao Paulo, Brazil | 6-14 | 60 (80%) | P-CPQ and FIS questionnaire by  one of the parent | Household crowding | Father’s education | Family income | Number of siblings Mother’s education | Moderate | [21] |
|  |  |  |  |  | Number of siblings | Family income |  |  |  |  |
|  |  |  |  |  | Mother’s education | Household crowding |  |  |  |  |
|  |  |  |  |  | Father’s education |  |  |  |  |  |
|  |  |  |  |  | Family income |  |  |  |  |  |
| CS | Children with cleft lip, University of Damascus, Syria | 6-14 | 87 (96.6%) | Modified CPQ_11-14_ questionnaire by  subjects facilitated by  interviews | Socio-economic  status scale  (based on education, occupation of  both the parents and monthly family income) | SES for all domains and overall CPQ_11-14_ score except  functional limitations | Not conducted | None | Weak | [26] |
| CS | Autistic children, one of their unaffected sibling and healthy children in Riyadh and Jeddah, Saudi Arabia | 8-13 | 59 families from  100 with autistic  children (59%)  matched  with 59 families  with no autistic  children | P-CPQ and FIS questionnaires  by any of the parent (translated and  validated in Arabic version in the same study but data not presented) | Mother’s age | Not done | For P-CPQ | For P-CPQ | Weak | [43] |
|  |  |  |  |  | Father’s age |  | Mother’s age | Father’s age |  |  |
|  |  |  |  |  | Father’s education |  | For FIS | Father’s education |  |  |
|  |  |  |  |  | Mother’s education |  | Mother’s age | Mother’s education |  |  |
|  |  |  |  |  | Family income |  | Mother’s  education | Total monthly income |  |  |
|  |  |  |  |  |  |  | Total monthly income | For FIS |  |  |
|  |  |  |  |  |  |  |  | Father’s age |  |  |
|  |  |  |  |  |  |  |  | Father’s education |  |  |
| CS | Children with AIDS attending Child Institute of the School of Medicine, University of Sao Paulo, Brazil | 10-15 | 88 (90.7%) | CPQ_11-14_  questionnaire b y subjects | House ownership | None on overall CPQ_11-14_ score | House ownership for oral symptoms domain | House crowding | Moderate | [40] |
|  |  |  |  |  | House crowding |  | Caregiver of the family for social wellbeing domain. |  |  |  |
|  |  |  |  |  | Caregiver of  the family |  |  |  |  |  |
| CS | Children with tooth agenesis and complete dentition in the clinics of Birminghan Dental Hospital, UK. | 11-14 | 86 with tooth  agenesis and 30 with complete dentition | CPQ_11-14_ (16 item) by children and  P-CPQ by parents | Social deprivation/ Socio-economic  status | None | Not conducted  for the effect of SES on OHRQoL | Socioeconomic status | Moderate | [32] |
| CS | Grade 6 and grade 8 school children in geographic areas served by Public Health Departments of York Region and Brant County, Canada | 11-14 | 370 | CPQ_11-14_  (10 item short form) questionnaire by children | Family dental insurance coverage | Family dental insurance coverage | Household  income | Number of children in the household (no data in results on tits effect on OHRQoL) | Weak | [36] |
|  |  |  |  |  | Number of adults in the household |  |  |  |  |  |
|  |  |  |  |  | Number of  children in the household | Number of adults in the household | Number of adults in the household |  |  |  |
|  |  |  |  |  | Household income | Household income |  |  |  |  |
|  |  |  |  |  | Receipt of  government income support | Receipt of government income support |  |  |  |  |
|  |  |  |  |  | Mother’s education | Mother’s education |  |  |  |  |
| CS | Public school children of Osorio, South Brazil | 11-14 | 515 (80.5%) | CPQ_11-14_  (16 item impact short form) questionnaire by subjects | Family structure (living with both parents/ living with only one parent or neither of them) | Family income | Family income | Mother’s education | Moderate | [47] |
|  |  |  |  |  | Mother’s education | Family structure | Family structure |  |  |  |
|  |  |  |  |  | Family income |  |  |  |  |  |
| CS | Public school children of Santa Maria, Brazil. | 11-14 | 944 (94%) | CPQ_11-14_  questionnaire by subjects | Mothers education | Household income  for overall CPQ  score, Oral  symptoms,  emotional and  social wellbeing | Household  income for  overall CPQ score, Oral symptoms, emotional and social wellbeing | Household income for functional limitation domain | Moderate | [51] |
|  |  |  |  |  | Fathers education | Mother’s  education for emotional well-being domain |  | Mother’s education for overall CPQ, Oral symptoms, functional limitations and social well-being domain |  |  |
|  |  |  |  |  | Household income |  |  | Father’s education on overall score and all domains |  |  |
| CS | Intermediate school children  of Dunedin, New Zealand | 12-13 | 354 (58.8%) | CPQ_11-14_  (16 item impact short form) questionnaire  by subjects | Area based  deprivation | Area based  deprivation | None | None | Weak | [29] |
| Data from two previous Cross-sectional studies | Intermediate  School children of Taranaki and Otago of New Zealand | 12-13 | 783 | CPQ_11-14_(16 item  impact short form) questionnaire  by subjects | Area based  deprivation as a measure of SES | None | None | Area based deprivation | Weak | [50] |
| Retrospective | Public school children of  city of Manaus, State of Amazonas, Brazil | 12-14 | 300 (77.1%) | Child-OIDP by interviewing the  subjects | Mothers age | Mother’s age | Financial government  support | Mother’s schooling, House crowding | Moderate | [25] |
|  |  |  |  |  | Mother’s schooling |  | Father’s  schooling | Family income |  |  |
|  |  |  |  |  | Father’s schooling |  | Mother’s age |  |  |  |
|  |  |  |  |  | Financial  governmental support |  |  |  |  |  |
|  |  |  |  |  | House crowding |  |  |  |  |  |
|  |  |  |  |  | Family income |  |  |  |  |  |
| Prospective study with 2 year  follow up | Children  attending orthodontics clinics and schools near Rio de Janeiro State University, Brazil | 12-15 | 284 (89.5%) | OHIP-14  questionnaire by subjects | Socio-economic  status (according to Brazil Economic Classification Criteria) | None | None | Socio-economic status | Strong | [28] |

OIDP - Oral Impacts on Daily Performance; CPQ - Child Perceptions Questionnaire; P-CPQ - Parental-Caregivers Perceptions Questionnaire; FIS - Family Impact Scale; OHIP - Oral Health Impact Profile.
